# A replacement name for *Dayus* Gerken, 2001 (Crustacea, Peracarida, Cumacea), preoccupied by *Dayus* Mahmood, 1967 (Insecta, Hemiptera, Cicadellidae)

**DOI:** 10.3897/zookeys.414.7673

**Published:** 2014-06-06

**Authors:** Yu-Bo Zhang, Sarah Gerken, Xiang-Sheng Chen

**Affiliations:** 1 Institute of Entomology, The Special Key Laboratory for Development and Utilization of Insect Resources, Guizhou University, Guiyang, Guizhou PR China, 550025; 2 Special and Key Laboratory of Functional Materials and Resource chemistry of Guizhou Provincial Education Department, Anshun University, Anshun, Guizhou PR China, 561000; 3 University of Alaska, Anchorage, 3211 Providence Dr., Biological Sciences, Anchorage, Alaska 99508, U.S.A.

**Keywords:** Crustacea, Peracarida, Cumacea, homonym, replacement name

## Abstract

A replacement name is proposed for genus *Dayus* Gerken, 2001 (Crustacea: Peracarida: Cumacea), preoccupied by *Dayus* Mahmood, 1967 (Insecta: Hemiptera: Cicadellidae). The following changes are proposed: *Jennidayus* **new replacement name** = *Dayus* Gerken, 2001 (nec Mahmood 1967); *Jennidayus pharocheradus* (Gerken, 2001), **comb. n.** = *Dayus pharocheradus* Gerken, 2001; *Jennidayus acanthus* (Gerken, 2001), **comb. n.** = *Dayus acanthus* Gerken, 2001; *Jennidayus makrokolosus* (Gerken, 2001), **comb. n.** = *Dayus makrokolosus* Gerken, 2001.

## Introduction

The cumacean genus name *Dayus* Gerken, 2001 (Crustacea: Peracarida: Cumacea) is preoccupied by *Dayus* Mahmood, 1967 (Insecta: Hemiptera: Cicadellidae). The purpose of the present paper is to propose a replacement name.

## Nomenclatural changes and notes

### Genus Jennidayus nom. n.

Dayus Gerken, 2001: 9 (Crustacea: Peracarida: Cumacea). Preoccupied by *Dayus* Mahmood, 1967: 39 (Insecta: Hemiptera: Cicadellidae).

#### Type species.

*Dayus pharocheradus* Gerken, 2001.

#### Remarks on nomenclatural change.

The cumacean genus *Dayus* was established by Gerken (2001) with three species: *Dayus pharocheradus*, *Dayus acanthus* and *Dayus makrokolosus* from Australia. *Dauys pharocheradus* was designated as the type species. However, *Dayus* Gerken, 2001 is preoccupied by *Dayus* Mahmood, 1967, established for a typhlocybine leafhopper genus, with *Dayus elongatus* Mahmood, 1967 (from Singapore) as the type species (Insecta: Hemiptera: Cicadellidae). According to Article 60 of the International Code of Zoological Nomenclature (ICZN 1999), we propose the new replacement name *Jennidayus* nom. n. for *Dayus* Gerken, 2001. Accordingly, three new combinations are herein proposed for the species currently included in this genus: *Jennidayus pharocheradus* (Gerken, 2001), *Jennidayus acanthus* (Gerken, 2001) and *Jennidayus makrokolosus* (Gerken, 2001).

#### Etymology.

The genus name was meant to honor Jennifer Day of South Africa, for her work on South African Cumacea; gender masculine.

#### Distribution.

Australia.

### Summary of nomenclatural changes

*Jennidayus* **new replacement name** = *Dayus* Gerken, 2001 (nec Mahmood, 1967).

*Jennidayus pharocheradus* (Gerken, 2001), **comb. n.** = *Dayus pharocheradus* Gerken, 2001.

*Jennidayus acanthus* (Gerken, 2001), **comb. n.** = *Dayus acanthus* Gerken, 2001.

*Jennidayus makrokolosus* (Gerken, 2001), **comb. n.** = *Dayus makrokolosus* Gerken, 2001.

## Supplementary Material

XML Treatment for Jennidayus

## References

[B1] Gerken S (2001) The Gynodiastylidae (Crustacea: Cumacea). Memoirs of Museum Victoria 59(1): 1–276

[B2] ICZN (1999) International Code of Zoological Nomenclature, fourth Edition The International Trust for Zoological Nomenclature, London, 306 pp

[B3] Mahmood SH (1967) A study of the Typhlocybinae genera of the Oriental Region (Thailand, The Philippines and adjoining areas). Pacific Insects Monograph 12: 1–52

